# Hf/Sb co-doping induced a high thermoelectric performance of ZrNiSn: First-principles calculation

**DOI:** 10.1038/s41598-017-15205-y

**Published:** 2017-11-06

**Authors:** Ju Zhang, Xiwen Zhang, Yuanxu Wang

**Affiliations:** Institute for Computational Materials Science, School of Physics and Electronics, Henan University, Kaifeng, 475004 People’s Republic of China

## Abstract

Previous experiments showed that Hf/Sb co-doping in ZrNiSn impressively improved the electrical conductivity (*σ*). To explore the physical reasons for this improvement, the electronic structures of Hf_x_Zr_1−x_NiSn_1−y_Sb_y_ (x = 0, 0.25, 0.5; y = 0, 0.02) have been systematically investigated by using the first-principles method and semiclassical Boltzmann transport theory. 50% Hf doping at Zr site in ZrNiSn simultaneously increases the degeneracy and dispersion of energy bands near the conduction band edge, which are helpful to optimizing Seebeck coefficient and slightly improving *σ*. Furthermore, 2% Sb co-doping at Sn site in Hf_0.5_Zr_0.5_NiSn not only increases total density of states near the Fermi energy but also retains high mobility, and *N*_*v*_ reaches eleven at the conduction band minimum, thereby inducing a large improvement in *σ*. Additionally, the Bader charge analysis shows the reason why Sb co-doping supplies more electrons. It is most likely derived from that Sb loses more electrons and Sb-Ni has a stronger hybridization than Sn-Ni. Moreover, we predict that the *ZT* of Hf_0.5_Zr_0.5_NiSn_0.98_Sb_0.02_ at 1000 K can reach 1.37 with the carrier concentration of 7.56 × 10^18^ cm^−3^, indicating that Hf/Sb co-doping may be an effective approach in optimizing thermoelectric properties of ZrNiSn alloy compounds.

## Introduction

Exploration of sustainable new clean energies has become a global consistent viewpoint, due to the depletion of fossil fuels and the resulting in environmental pollution^1^. Thermoelectric (TE) materials can perform directly converting waste heat into electricity using in all-solid-state by a temperature difference to induce carriers to flow in a semiconductor, which are helpful to resolving today’s energy crisis^2–4^. Although the TE devices are very reliable and compact, the relatively low efficiency limits their widespread applications. The conversion efficiency of a TE material can be characterized by the dimensionless figure of merit *ZT*, defined as *ZT* = *S* ^2^ *σT*/(*κ*_*e*_ + *κ*_*l*_), where *S*, *σ*, *T*, *κ*_*e*_, and *κ*_*l*_ are the Seebeck coefficient, electrical conductivity, absolute temperature, electrical thermal conductivity, and lattice thermal conductivity, respectively^5–7^. An excellent TE material should have a large *S*, a high *σ*, and a low *κ*_*l*_, comparatively speaking a high *κ*_*e*_ is not a problem^8–10^.

For metals or degenerate semiconductors^11^, *S*, density-of-states effective mass (${m}_{{\rm{DOS}}}^{\ast }$), *σ*, and carrier mobility (*μ*) are given by^5,12^:

1 $$S=\frac{8{\pi }^{2}{{k}_{B}}^{2}}{3e{h}^{2}}{m}_{{\rm{DOS}}}^{\ast }T{(\frac{\pi }{3n})}^{\mathrm{2/3}},$$

2 $${m}_{{\rm{DOS}}}^{\ast }={N}_{v}^{\mathrm{2/3}}{m}_{{\rm{b}}}^{\ast },$$

3 $$\sigma =ne\mu ,$$

4 $$\mu \propto {m}_{{\rm{b}}}^{\ast -\mathrm{5/2}},$$

where ${m}_{{\rm{b}}}^{\ast }$ is the band effective mass and *n* is the carrier concentration. From the above formulas, we can clearly see that *S* and *σ* have an inverse dependence on *n*. A large ${m}_{{\rm{b}}}^{\ast }$ is favorable to enlarge *S* by increasing the ${m}_{{\rm{DOS}}}^{\ast }$ while it will in turn lead to a significant reduction in *σ* via *μ* ^13,14^. Therefore, it is imperative to achieve the balance between *σ* and *S* via optimizing *n* and adjusting ${m}_{{\rm{b}}}^{\ast }$ to maximize power factor (*S* ^2^ *σ*). For this purpose, it is valuable to find an appropriate extrinsic dopant to provide a high *n* with a low deformation potential (Ξ) and a low alloy scattering potential^15–17^. A low Ξ and a low alloy scattering potential, which signify a weak electron-phonon interaction, are favorable for obtaining a high *μ*. Considering low scattering is not conducive to suppressing *κ*_*l*_, therefore an appropriate dopant inducing multiscale scattering centers is also vital by making a compromise between *μ* and *κ*_*l*_ for attaining high TE performance.

Obeying 18-valence-electron rule, ZrNiSn has a cubic structure and a narrow band gap (*E*_g_ ~ 0.5 eV) with relatively large *S* (~213 V K^−1^ at 800 K) and high structural stability even at high temperature^18^. However, the *κ* of pure ZrNiSn is high and its *σ* is small (~5.85 × 10^4^ Ω^−1^ *m* ^−1^ at 800 K), which lead to its low *ZT* value. The nanostructuring strategy has been employed to reduce *κ*_*l*_ by enhancing phonon scattering at the grain boundaries, but it will deteriorate *μ*, which especially limits the improvement of *ZT*. To overcome this limitation, the isoelectronic substitution of Hf at Zr site in ZrNiSn has been proved to be highly effective to reduce *κ*_*l*_ and optimize electrical properties^19,20^. Previous experiments showed that 50% than 25% Hf doping in ZrNiSn could more efficiently enhance TE performance, which not only increased *S* (~252 *μ*V K^−1^ at 800 K), but also slightly increased *σ* (~5.9 × 10^4^ Ω^−1^ *m* ^−1^ at 800 K)^21–24^. Those experimental works have detailedly discussed the supression of *κ*_*l*_, however, there is no specific analysis of the reasons for the increasing of power factor (PF) of Hf_x_Zr_1−x_NiSn (x = 0.25, 0.5). Therefore, it is necessary to devote more effort to analyzing the physical reason of improved PF. Although Hf doping increases *S*, there is a little increase in *σ*. Hence, it is necessary to further optimize *σ* for higher TE properties. Yu *et al*.^22^ reported that the *κ*_*l*_ of Hf_x_Zr_1−x_NiSn_0.98_Sb_0.02_ (x = 0.5, 0.6) samples reduced to 3.1 W m^−1^ K^−1^ and 3.3 W m^−1^ K^−1^ at room temperature, respectively, and n-type 2% Sb doping at Sn site in Hf_0.5_Zr_0.5_NiSn has shown to be an effective approach to further remarkably enhance *σ* (~14.8 × 10^4^ Ω^−1^ *m* ^−1^ at 800 K) by optimizing *n*. Nevertheless, they also did not explain why Sb co-doping in Hf_0.5_Zr_0.5_NiSn could further efficiently improve TE properties.

In this work, to explore the influences of Hf/Sb co-doping, we substituted Zr sites with various Hf doping contents and co-doping Sb at Sn site in ZrNiSn, and systematically investigated the electronic structures and transport properties of Hf_x_Zr_1−x_NiSn_1−*y*_Sb_y_ (x = 0, 0.25, 0.5; y = 0, 0.02) by using the first-principles calculations and semiclassical Boltzmann transport theory. Our calculation results show that 50% Hf doping in ZrNiSn simultaneously increases the degeneracy (*N*_*v*_) at the bottom of conduction band (CB) and the dispersion of energy-band near the CB edge, which are helpful to increasing *S* and slightly enhanceing *σ*. Meanwhile, we elucidate that why 2% Sb co-doping further largely improves *σ*. It increases total density of states near the Fermi energy (*E*_F_) and leads to the convergence of the light and heavy bands and valley degeneracy. *N*_*v*_ reaches eleven at the Γ point of the bottom of conduction band. Besides, our work also demonstrates that Hf_0.5_Zr_0.5_NiSn_0.98_Sb_0.02_still maintains high *μ* in spite of ${m}_{{\rm{b}}}^{\ast }$ increasing. Therefore, a great improvement in *σ* occurs. The *ZT* (~1.37) of Hf_0.5_Zr_0.5_NiSn_0.98_Sb_0.02_ with the *n* of 7.56 × 10^18^ cm^−3^ at 1000 K is predicted.

## Results and Discussion

### Crystal structure and bonding properties of pure and Hf/Sb co-doped ZrNiSn

**Figure 1:**
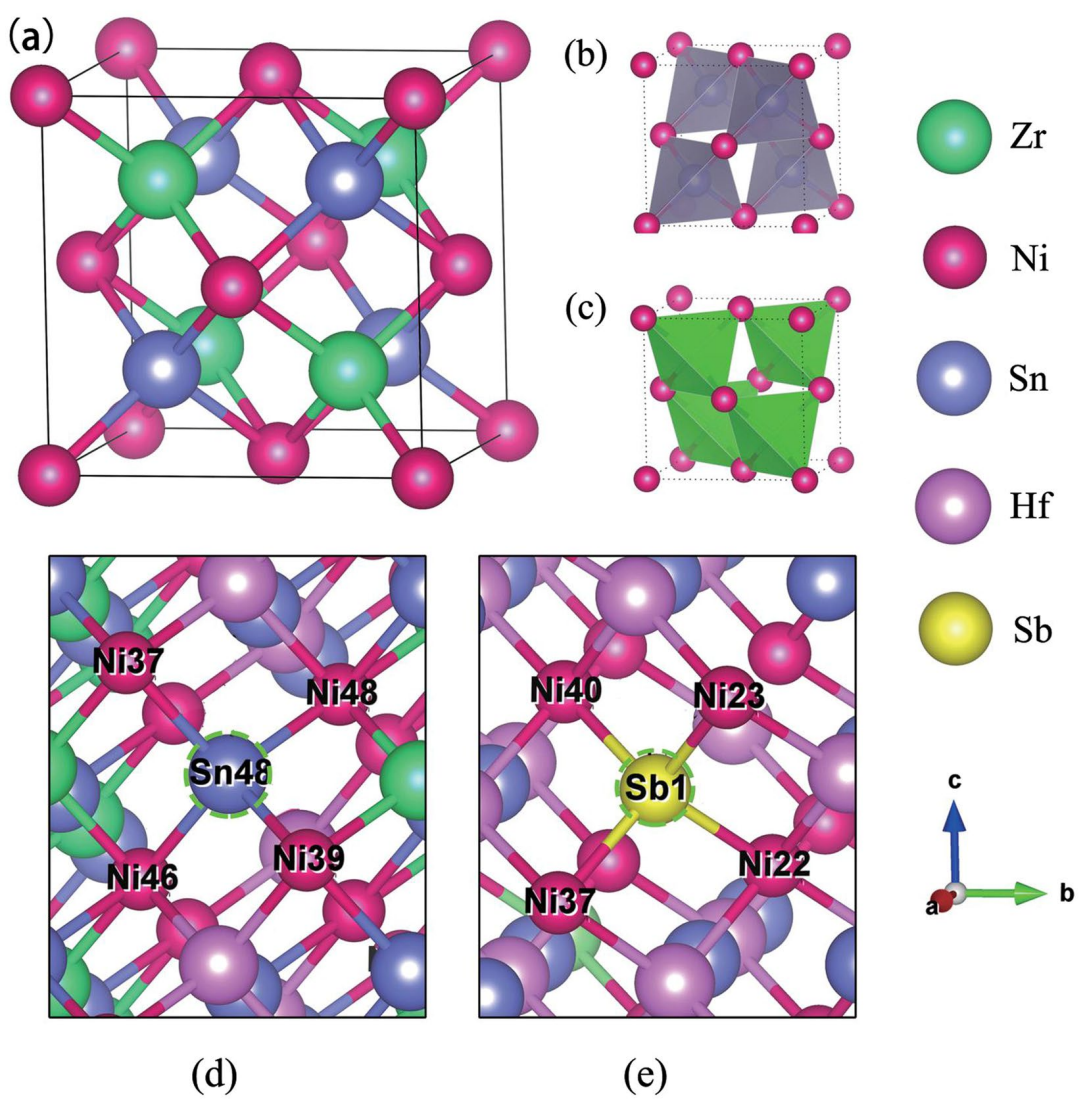
(**a**) The crystal structure of half-Heusler ZrNiSn with the space group of $F\bar{4}3\,m$, no. 216. Corner sharing of (**b**) SnNi_4_ and (**c**) ZrNi_4_ tetrahedra. (**d**) Local structure of Hf_0.5_Zr_0.5_NiSn. (**e**) Local structure of Hf_0.5_Zr_0.5_NiSn_0.98_Sb_0.02_.

The C1_*b*_-type structure of ZrNiSn (space group: F $\bar{4}3\,m$, no. 216)^25^ is crystallized by three interpenetrating facecentered cubic (fcc) sublattices^26^, as depicted in Fig. 1(a). Each unit cell contains four Zr atoms, four Ni atoms, and four Sn atoms. The electronegativity values of Zr, Ni, and Sn are 1.33, 1.91, and 1.96, respectively. The most electropositive element Zr donates all of its valence electrons to the more electronegative elements Ni and Sn, as a result, ZrNiSn can be described as Zr^4+^ (NiSn)^4−^ ^27^. This system is filled by the substructures which are similar to ZnS  lattice. These substructures are formed by Ni atoms with Sn or Zr in the centers of the tetrahedron, as shown in Fig. 1(b,c). Figure 1(d,e) present the local structures of 50% Hf substitutions at Zr sites in a 2 × 2 × 3 ZrNiSn supercell and 2% Sb co-doping at Sn48 site in a 144-atoms cell of Hf_0.5_Zr_0.5_NiSn.

**Figure 2:**
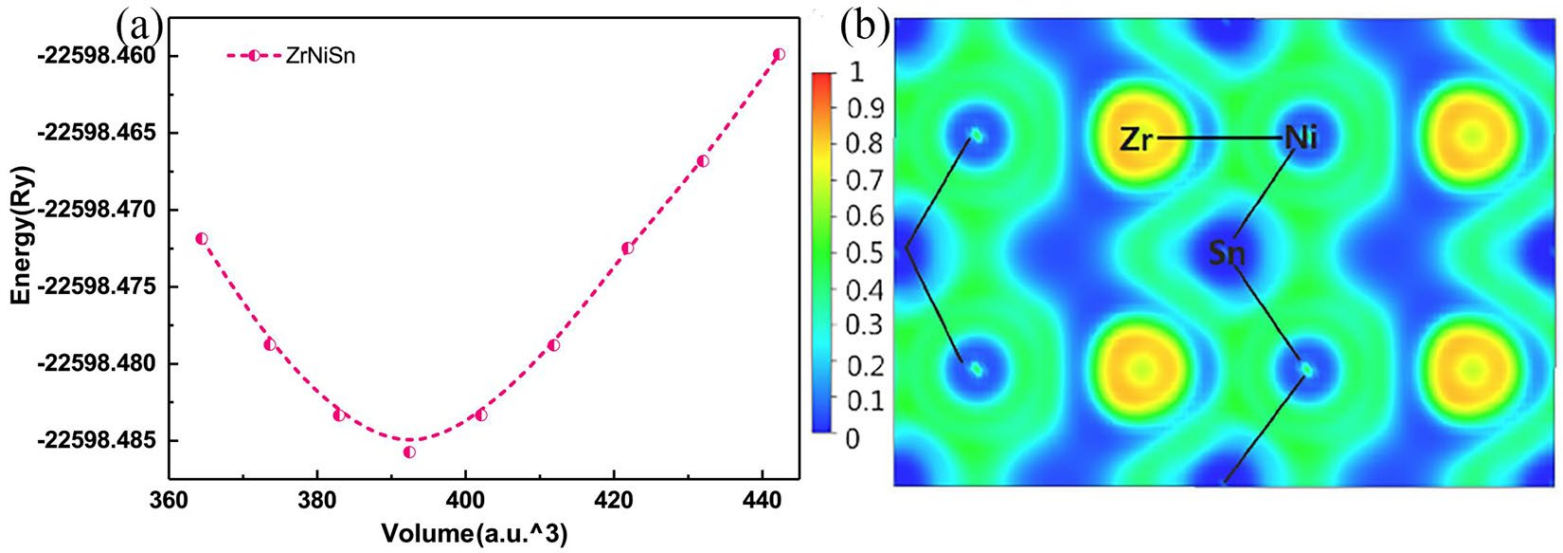
(**a**) The fitted E-V curve of ZrNiSn structure. (**b**) Calculated ELF in the (0 0 1) plane of ZrNiSn.

Using the equation of state (EOS) to fit the E-V curve, where E is the total energy of one unit cell (with the unit of Ry), V is the volume of one unit cell (with the unit of a.u.^3^), we firstly acquire the most stable ZrNiSn structure. Its lattice constant (*a*) is ~6.141 Å which is consistent with the experimental value (*a* ~ 6.110 Å), proving the reliability of our theoretical method. For well understanding of the electron distribution and bonding properties of ZrNiSn, we calculated the electron localization function (ELF)^28^. Figure 2(b) reveals that ELF is evenly distributed around Zr atoms, this means that Zr donates its valence electrons to the [NiSn]^4−^, which coincides with the above analysis that, in ZrNiSn, Zr atoms tend to lose electrons, while Ni and Sn atoms tend to gain electrons owing to their larger electronegativity. Whereas the ELF between the Zr and Ni atoms mainly localizes around Ni, and a certain number of charges appear in center position between Sn and Ni, indicating Zr and Ni atoms incline to form an ionic bond, while there is a distinct covalent bond characteristics between Ni and Sn atoms.

### Electronic structure analysis of Hf_x_Zr_1−x_NiSn_1−y_Sb_y_

**Figure 3:**
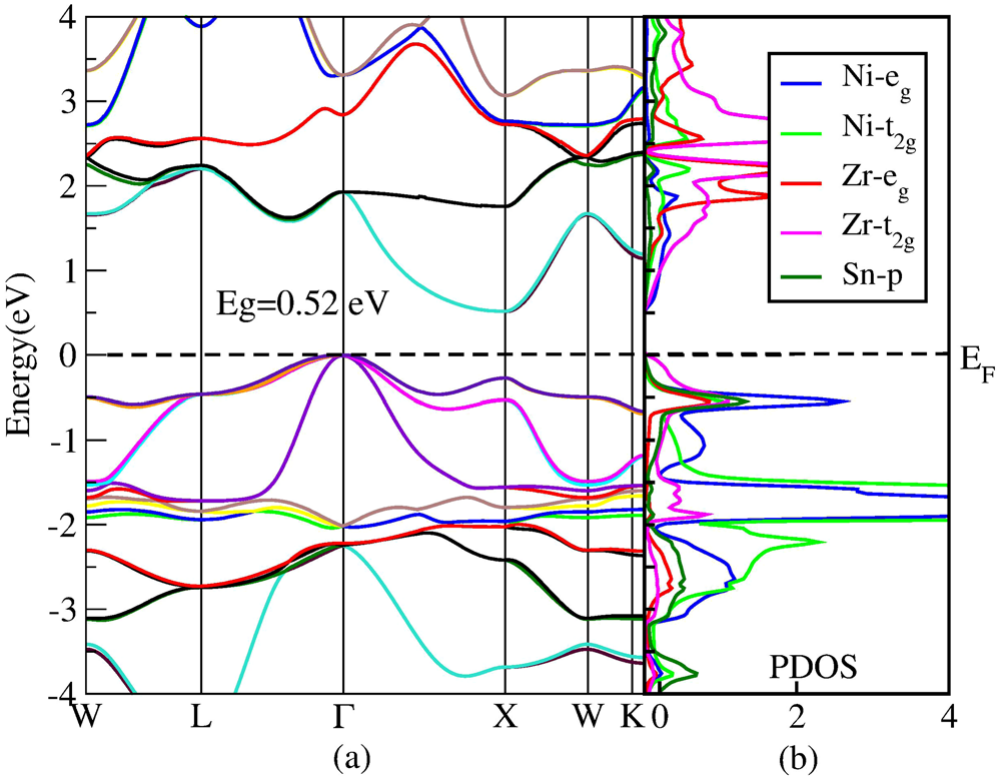
(**a**) Band structure of ZrNiSn. (**b**) Projected PDOS of ZrNiSn.

Regarding the transport properties are predominantly affected by the electronic states at the vicinity of the valence band maximum (VBM) and the conduction band minimum (CBM), we only concern with the electronic states near the *E*_F_. Usually good TE materials are narrow-band-gap semiconductors and precise *E*_g_ is very vital to estimating the TE performance. One important feature of the electronic structure is its band structure which is closely related to the TE properties of the materials. Figure 3(a,b) plots the band structure and partial density of states (PDOS) of ZrNiSn. With modified mBJ method by DFT, we calculated the band structure of ZrNiSn. The calculated *E*_g_ of ZrNiSn is about 0.52 eV which is similar to that with PBE-GGA (0.51 eV), and it is also good agreement with previously calculated value (~0.50 eV)^29,30^. The band gap can be estimated using experimental data from the temperature reliance on *σ* by^31^:

5 $$\sigma ={\sigma }_{0}\exp (-\frac{E{\rm{g}}}{2{k}_{B}T}),$$

where *σ*_0_ is a pre-exponential factor. Muta *et al*.^32^ estimated that the *E*_g_ of ZrNiSn is about 0.20 eV corresponding to the prior experimental value (~0.186 eV)^33,34^. It is extremely unexpected because DFT usually underestimates, not overestimates, *E*_g_. The *E*_g_ of experimental value is smaller than calculated value, which may originate from the formation of impurity band in the gap, due to the presence of Ni interstitial atoms with its *d* orbitals when Ni atoms excess (>25%), namely Frenkel pairs^35^. Despite a drastically reduced *E*_g_ due to Ni interstitial band^36^, the n-type ZrNiSn is not over-whelmed by bipolar effect, because the holes in ZrNiSn have low *μ* ^37^, leading to a low hole electrical conductivity (*σ*_*p*_). As a result of the total *S* for multiple carriers is weighted by the individual *σ* ^38^:

6 $${S}_{{\rm{total}}}=({S}_{{\rm{n}}}{\sigma }_{{\rm{n}}}+{S}_{{\rm{p}}}{\sigma }_{{\rm{p}}})/({\sigma }_{{\rm{n}}}+{\sigma }_{{\rm{p}}}\mathrm{).}$$

This is one possible reason why ZrNiSn normally has a relatively large *S*. The *N*_*v*_ at the Γ point of the top of valence band (VB) is six, this relatively high *N*_*v*_ is the other possible reason for having a relatively large *S*. It is well known that *ZT* inversely depends on *κ*. Therefore, the optimal dopants not only can substantially improve PF, but also can significantly suppress *κ*.

**Figure 4:**
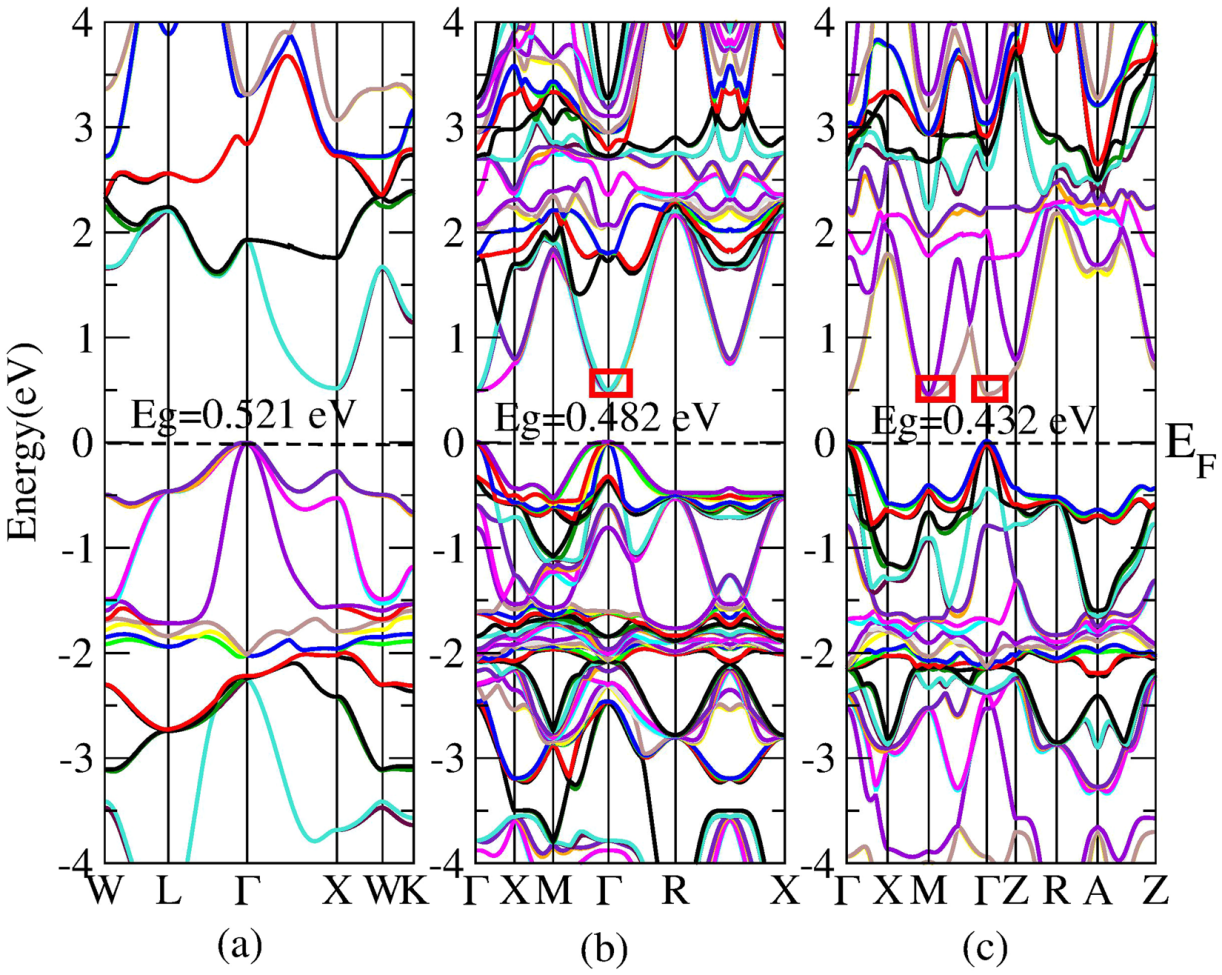
Calculated band structures of ZrNiSn (**a**), Hf_0.25_Zr_0.75_NiSn (**b**), and Hf_0.5_Zr_0.5_NiSn (**c**).

**Figure 5:**
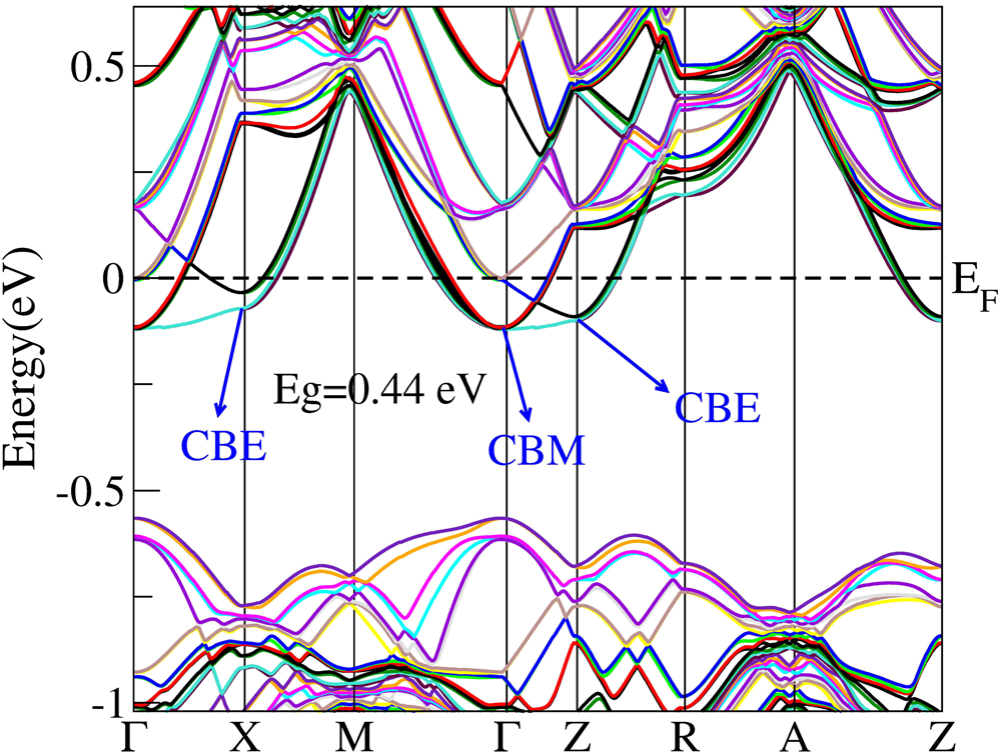
Band structure of Hf_0.5_Zr_0.5_NiSn_0.98_Sb_0.02_. High symmetry k points Γ, X, M, Z, R and A in the figure represent the points (0, 0, 0), (0, 0.5, 0), (0.5, 0.5, 0), (0, 0, 0.5), (0, 0.5, 0.5) and (0.5, 0.5, 0.5), respectively.

Previous experiments reported that Hf substitution at Zr site in ZrNiSn could significantly suppresse *κ*_*l*_ and improve electron transport simultaneously^19,20^. To clearly analyze the reason for this increase of PF, we also calculated the band structures of HfNiSn and Hf_x_Zr_1−x_NiSn (x = 0.25, 0.5). The band structure of HfNiSn was ploted in Supplemental Fig. S1. What is worth mentioning that all our calculations include the spin-orbital coupling (SOC). As has been reported that SOC is only a small effect on the band structure of ZrNiSn, while there is a distinct effect on the band structure of HfNiSn if SOC is included. That is why SOC is considered here. According to Eq. (2) we know, a high *N*_*v*_ will increase ${m}_{{\rm{DOS}}}^{\ast }$ and thus enlarges *S*, because which is proportional to ${m}_{{\rm{DOS}}}^{\ast }$. *N*_*v*_ contains the orbital degeneracy (*N*_*v*_ at one extrema point) and valley degeneracy (separate pockets at the same or similar energy). As shown in Fig. 4(a), the *N*_*v*_ of ZrNiSn at the X point of the bottom of CB is 2. While we can note from Fig. 4(b) that 25% Hf doping in ZrNiSn makes six orbital degeneracy at the Γ point of the bottom of CB, which may increase its *S*. Figure 4(c) depicts that 50% Hf doping induces six effective electron-pockets with the difference in energy between them is 0.013 eV at the M point (*N*_*v*_ = 4) and Γ point (*N*_*v*_ = 2) of the bottom of CB. This means that the energy bands of M and Γ points of the bottom of CB simultaneously participate in transport, which may be responsible for the improvement of *S* of Hf_0.5_Zr_0.5_NiSn. Yu *et al*. reported that the n-type 2% Sb co-doping at Sn site in Hf_0.5_Zr_0.5_NiSn could further greatly improve *σ* ^22^. To explore physical reason for such improvement, we calculated the band structure of Hf_0.5_Zr_0.5_NiSn_0.98_Sb_0.02_. *N*_*v*_ increases when the difference in energy of their band extrema within a few *k*_*B*_ *T*, and *σ* enhances. Because in a system, the total electrical conductivity (*σ*_*total*_) can be expressed as:

7 $${\sigma }_{total}={\sigma }_{1}+{\sigma }_{2}$$

when it contains two valence (or conduction) bands. Here, subscripts 1 and 2 refer to the transport properties of carriers in the individual band^39^. As seen in Fig. 5, there are eleven approximately degenerated bands (*N*_*v*_ = 11) at the Γ point of the bottom of CB, which are conducive to improving *σ*_*total*_. This also makes *S* not fall too much via enlarging ${m}_{{\rm{DOS}}}^{\ast }$ as governed by Eq. (2), this is one of the possible reasons why *σ* has been impressively enhanced without reduction *S* too much. Apart from the CBM, there are two other conduction band extremums (CBEs) with little difference in energy, one occurs at the X point, and another lies in the Z point. These conduction valleys have almost equal energies, as shown in Table 1, therefore increasing valley degeneracy. The ${m}_{{\rm{DOS}}}^{\ast }$ can be re-written as^40^:

8 $${m}_{{\rm{DOS}}}^{\ast }={({N}_{1}{m}_{1}^{\ast \mathrm{3/2}}+{N}_{2}{m}_{2}^{\ast \mathrm{3/2}}+{N}_{3}{m}_{3}^{\ast \mathrm{3/2}})}^{\mathrm{2/3}},$$

where *N*_1_, *N*_2_ and *N*_3_ are the valley degeneracies for the CBM and two CBEs, respectively. According to Eq. (7), the higher valley degeneracy of the CBM and CBEs are favorable for higher *σ*_*total*_ and larger ${m}_{{\rm{DOS}}}^{\ast }$. This is another possible reason for impressively enhacing *σ* and no too much reduction of *S*. Meanwhile, at the Γ point, the light band with small ${m}_{{\rm{b}}}^{\ast }$ (~0.63 *m*_e_) at the vicinity of the *E*_F_ is beneficial to enhancing *σ* and the heavy band with large ${m}_{{\rm{b}}}^{\ast }$ (~4.23 *m*_e_) close to the CBM is conducive to *S*. The coexistence of light and heavy bands, accompanying by an increase in the number of effective electron-pocket near the *E*_F_, may strongly increase *σ* of Hf_0.5_Zr_0.5_NiSn_0.98_Sb_0.02_ without too much decreasing *S*. The remarkable advantage of this band structure is the band convergence induced by 2% Sb doping, which increases *ZT* value in the whole temperature range. Figure 5 presents that n-type 2% Sb co-doping makes Hf_0.5_Zr_0.5_NiSn_0.98_Sb_0.02_ become a degenerate semiconductor with *E*_F_ moving into CB by 0.12 eV. The large *n* and the appearance of light band near the *E*_F_ may remarkably enhance *σ*, in the meantime, it also probably decreases *S*. While the heavy band and high *N*_*v*_ are favorable for increasing *S*, which will overwhelm detriment from increasing *n*, thus that’s why *σ* dramatically increases without too much decreasing *S*.

**Table 1:** The energy eigenvalues (in unit of eV) of CBM and two CBEs of Hf_0.5_Zr_0.5_NiSn_0.98_Sb_0.02_.

CBM (Γ point)	CBE (X point)	CBE (Z point)
−0.115	−0.072	−0.112

**Figure 6:**
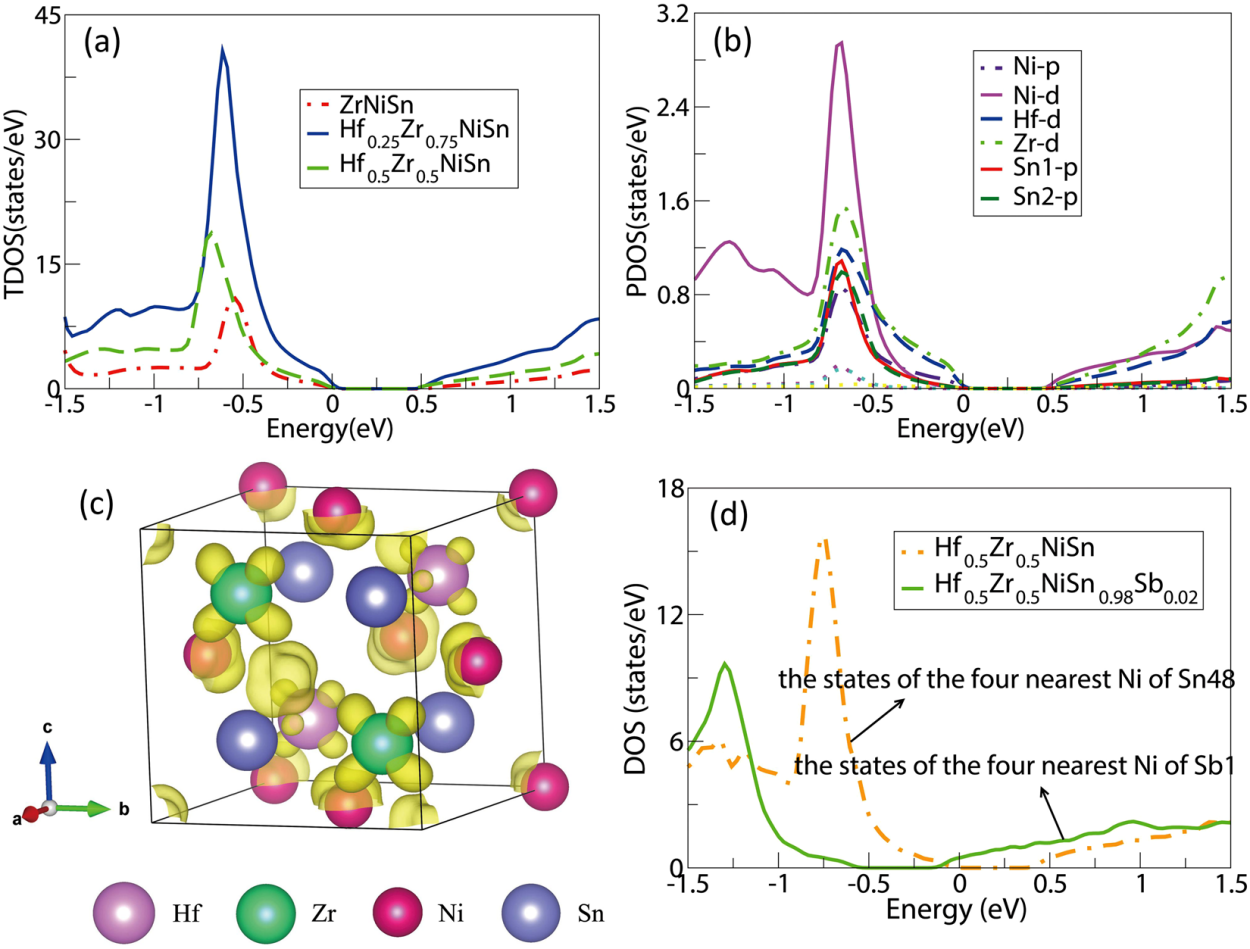
(**a**) Calculated the TDOS of ZrNiSn, Hf_0.25_Zr_0.75_NiSn and Hf_0.5_Zr_0.5_NiSn. (**b**) PDOS of Hf_0.5_Zr_0.5_NiSn. (**c**) The band decomposition charge density of Hf_0.5_Zr_0.5_NiSn at the bottom of CB with the isosurface value of 0.003 e/Å^3^. (**d**) The states of the four nearest Ni atoms of Sn48 and Sb1.

**Table 2:** The total number of atoms (*N*), the relaxation time at 700 K, the average energy band effective mass near the CBM of ZrNiSn, Hf_0.5_Zr_0.5_NiSn and Hf_0.5_Zr_0.5_NiSn_0.98_Sb_0.02_ compounds.

Compound	*N*	*τ*	${\bar{{\boldsymbol{m}}}}_{{\boldsymbol{b}}}^{{\boldsymbol{\ast }}}$
ZrNiSn	12	3.10fs	1.09m_*e*_
Hf_0.25_Zr_0.75_NiSn	12	10.80fs	1.97m_*e*_
Hf_0.5_Zr_0.5_NiSn	12	9.88fs	1.18m_*e*_
Hf_0.5_Zr_0.5_NiSn_0.98_Sb_0.02_	144	14.4fs	2.31m_*e*_

To clearly understand the states near the *E*_F_, we calculated the PDOS of ZrNiSn. In Fig. 3(b), the band edge is mainly contributed by the Zr-t_2g_, and the bottom of the CB partly comes from Ni-e_g_. Thus, the substitution on the Zr site can effectively adjust the band structure near the band edge. Doping often results in reduction of *S*, however, Hf doping increases *S*. More particularly, 50% Hf doping simultaneously increases *S* and *σ*. To analyze the reasons for enhanced PF by Hf doping, we calculated the total density of states (TDOS) of Hf_x_Zr_1−x_NiSn (x = 0, 0.25, 0.5) as shown in Fig. 6(a). Hf doping increases the TDOS near the *E*_F_, in fact, an enhanced TDOS near the *E*_F_ contributes to forming a high *σ* due to *σ*(*E*_F_) ∝ TDOS${|}_{E={E}_{{\rm{F}}}}$. It is also found that the TDOS of x = 0.25 is larger than that of x = 0.5 near the band edge. While the larger ${m}_{{\rm{b}}}^{\ast }$ (~1.97 m_e_) of x = 0.25, as depicted in the Table 2, will reduce the *μ* than of x = 0.5, which may counteract the increase of *σ* by a larger TDOS. Moreover, 50% Hf doping also increases the dispersion of energy bands near the CB edge, and its smaller ${m}_{{\rm{b}}}^{\ast }$ (~1.18 *m*_e_) than x = 0.25 gives obvious evidence to this, which is also helpful to improving *σ*. As a consequence, 50% Hf doping can increase PF through simultaneously increasing *S* and slightly optimizing *σ*.

To further analyze the reasons for enhanced more PF by 50% Hf doping, we also calculated the partial DOS (Fig. 6(b)) and band decomposition charge density (Fig. 6(c)) of CBM and VBM of Hf_0.5_Zr_0.5_NiSn system. Figure 6(b) indicates that the contributions to energy band edge of both CB and VB are mainly comprised of *d* orbitals of Ni, Zr and Hf, and the corresponding ${m}_{{\rm{b}}}^{\ast }$ is large due to localizing 3*d* states. This fact is beneficial to electrical transport with increasing ${m}_{{\rm{DOS}}}^{\ast }$. Therefore, Hf_0.5_Zr_0.5_NiSn has a relatively large *S*. From Fig. 6(c), we can see that the charges accumulate around the Ni, Hf, and Zr atoms, while charges around Sn1 and Sn2 atoms are few at the CBM. Thereby Sb doping at Sn site may effectively increase the TDOS near the *E*_F_. Previous experiments showed that Sb doping at Sn site would further remarkably enhance *σ* without significantly reducing *S* ^22^. This motivates our great interest to investigate the reason for such improved TE properties. As seen in Fig. 6(d) that Sb doping leads to an increase in the TDOS near the CB edge, which are why *σ* remarkably improves.

### Enhancement of electrical transport properties by Hf/Sb co-doping in ZrNiSn

**Figure 7:**
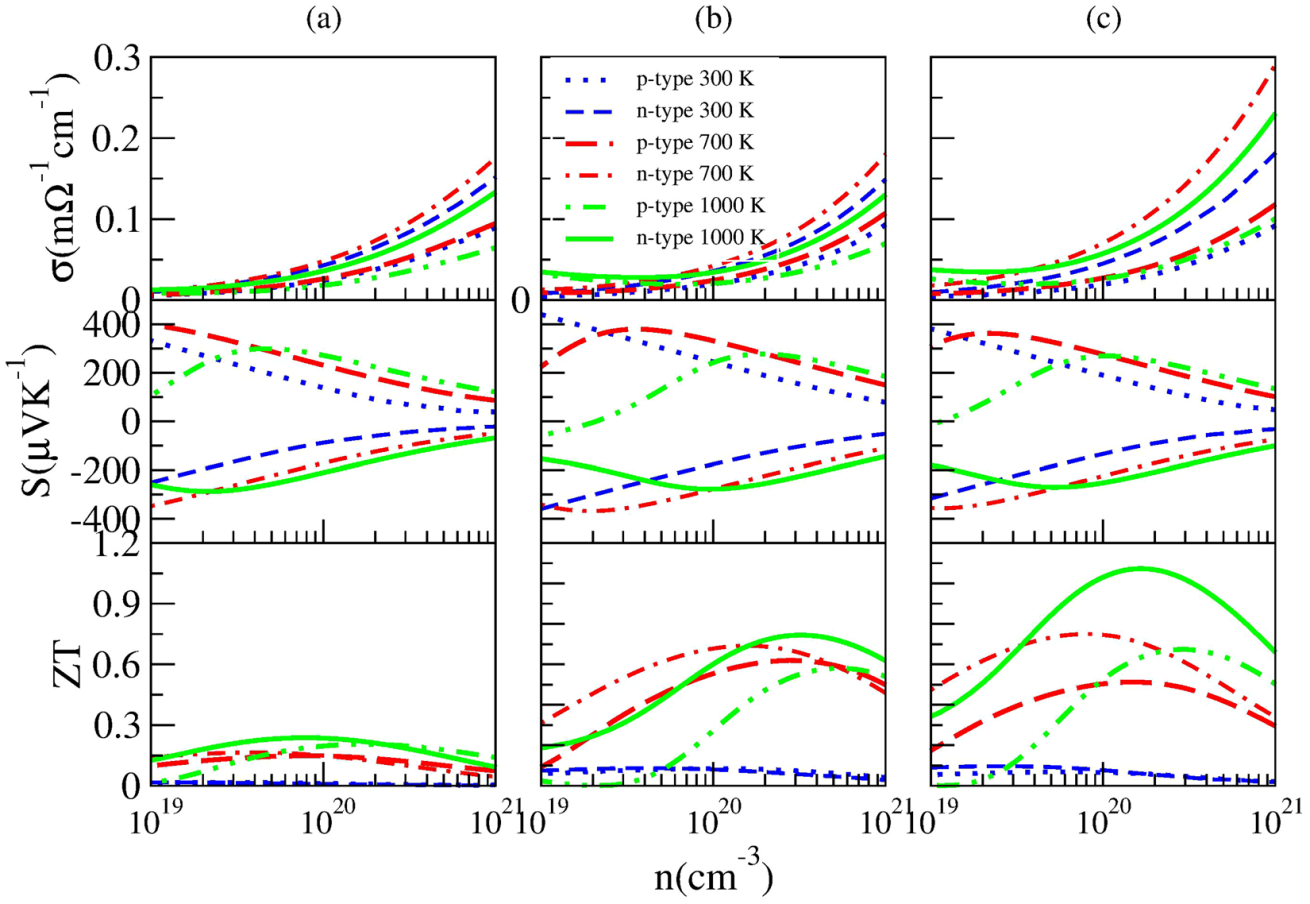
Calculated transport properties of *p*-type and *n*-type ZrNiSn (**a**), Hf_0.25_Zr_0.75_NiSn (**b**), and Hf_0.5_Zr_0.5_NiSn (**c**), respectively.

As mentioned above, a promising TE material requires a large *S*, a high *σ*, and a low *κ*. Experiments have shown that Hf substitution at Zr site in ZrNiSn can effectively reduce *κ*_*l*_ by isoelectronic substitution creating multiscale scattering centers and alloy scattering of phonons due to the mass and size differences between dopant atoms and host atoms^19,20^. Prior experiments demonstrated that Hf doping also enhanced PF and Sb doping in Hf_0.5_Zr_0.5_NiSn further primarily improved *σ* ^22^. To deeply understand the influences of Hf/Sb co-doping, herein various doping concentrations were simulated and the transport properties of Hf_x_Zr_1−x_NiSn_1−y_Sb_y_ (x = 0, 0.25, 0.5; y = 0, 0.02) were calculated as a function of *n* at 300 K, 700 K, and 1000 K within the framework of the semiclassical Boltzmann transport theory. The strategy previously used by Ong *et al*.^41^ was adopted with available experimental data^18–20,22^ to roughly estimate relaxation time (*τ*). Accordingly, the standard electron-phonon dependence on *T* and *n* for *τ* is: *τ* = *C*_0_ *T* ^−1^ *n* ^−1/3^ with *τ* in s, *T* in K and *n* in cm^−3^. For comparision the doping effect of Hf at different concentrations, we firstly calculated the electrical transport properties of Hf_x_Zr_1−x_NiSn (x = 0, 0.25, 0.5), as ploted in Fig. 7. Compared with pure phase of ZrNiSn (*ZT* ~ 0.24 with the optimal *n* of 7.745 × 10^19^ cm^−3^ at 1000 K) (Fig. 7(a)), 25% Hf doping increases *S* and almost unchanges *σ*, hence increasing the *ZT* (~0.74 with the optimal *n* of 3.162 × 10^20^ cm^−3^ at 1000 K) (Fig. 7(b)). Fascinatingly, 50% than 25% Hf doping can more effectively enhance the *ZT* (~1.07 with the optimal *n* of 1.710 × 10^20^ cm^−3^ at 1000 K) by synergistically enhancing *S* and *σ* (Fig. 7(c)). Herein, the *κ*_*l*_ of experimental value was employed for evaluating *ZT* ^18,19,22,42^. The calculated results also show that the TE properties of n-type Hf_x_Zr_1−x_NiSn (x = 0, 0.25, 0.5) compounds are better than those of p-type ones. This can be well explained by a large electronegativity difference (Δ*χ*) between Hf^4+^ and (NiSn)^4−^, which gives rise to a higher formation energy of cation antisite defects (*E*_AS_) and a lower formation energy of anion vacancies (*E*_*v*_)^8^.

**Figure 8:**
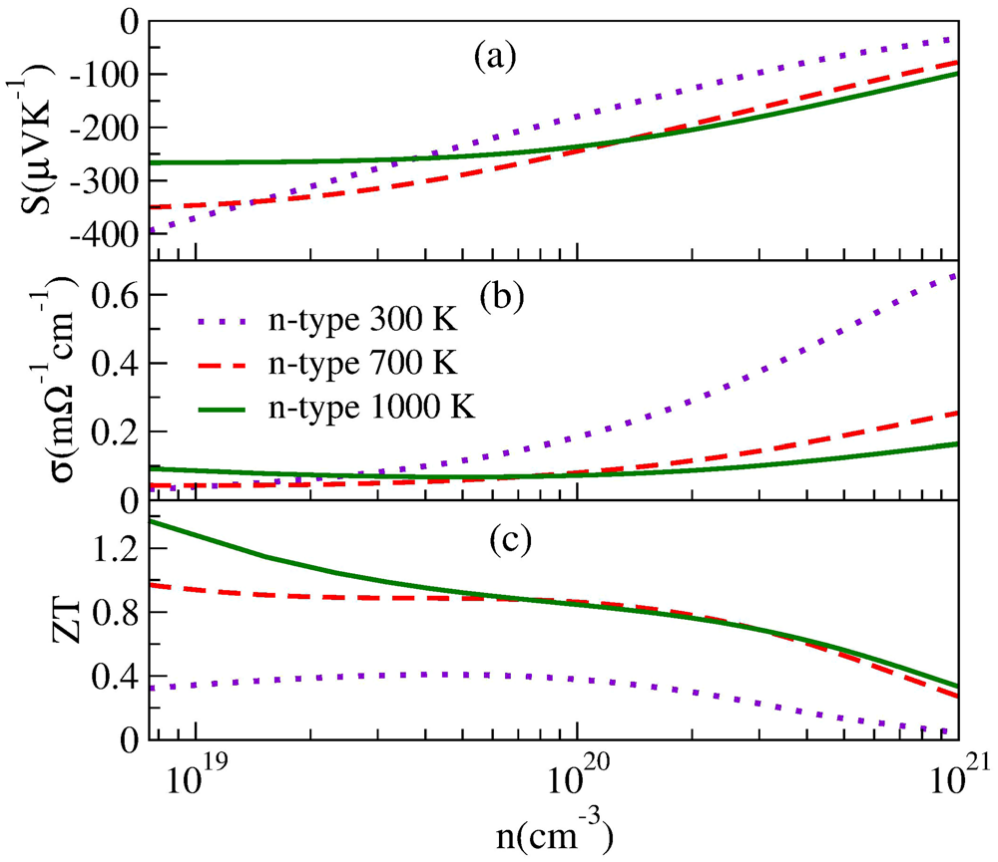
Calculated electrical transport coefficients of Hf_0.5_Zr_0.5_NiSn_0.98_Sb_0.02_ as a function of carrier concentration at 300 K, 700 K, and 1000 K.

To further research the effect of Sb co-doping, we calculated the electrical transport properties of Hf_0.5_Zr_0.5_NiSn_0.98_Sb_0.02_ by combining the semiclassical Boltzmann transport theory under constant *τ* approximation. As shown in Fig. 8, the peak *ZT* can reach 1.37 by achieving strikingly improvement of *σ* and no too much reduction of *S* with the *n* of 7.56 × 10^18^ cm^−3^ at 1000 K. It should be noted that the experiment workers only measured the *κ* before 800 K^22^. While calculating *κ* of large doping systems are usually difficult. Here, the *κ* of Hf_0.5_Zr_0.5_NiSn_0.985_Sb_0.015_^20^, which probably has a similar crystal structure to Hf_0.5_Zr_0.5_NiSn_0.98_Sb_0.02_ and thereby has analogous *κ*, was adopted to predict the *ZT* of Hf_0.5_Zr_0.5_NiSn_0.98_Sb_0.02_ at 1000 K. From Fig. 8(a), we can see that *S* is affected by bipolar effect decreasing at high temperature. This reduction can be attributed to the convergence of light and heavy bands at the bottom of CB. The bipolar effect may be caused by the small direct *E*_g_ (~0.44 eV) as displayed in Fig. 5, in which the excitation of electron-hole pairs and the opposing contribution to *S*_total_ from the two types of carriers^23^. To our knowledge, the bipolar effect will decrease the TE performance, thus it is valuable to seek method for decreasing bipolar effect.

**Figure 9:**
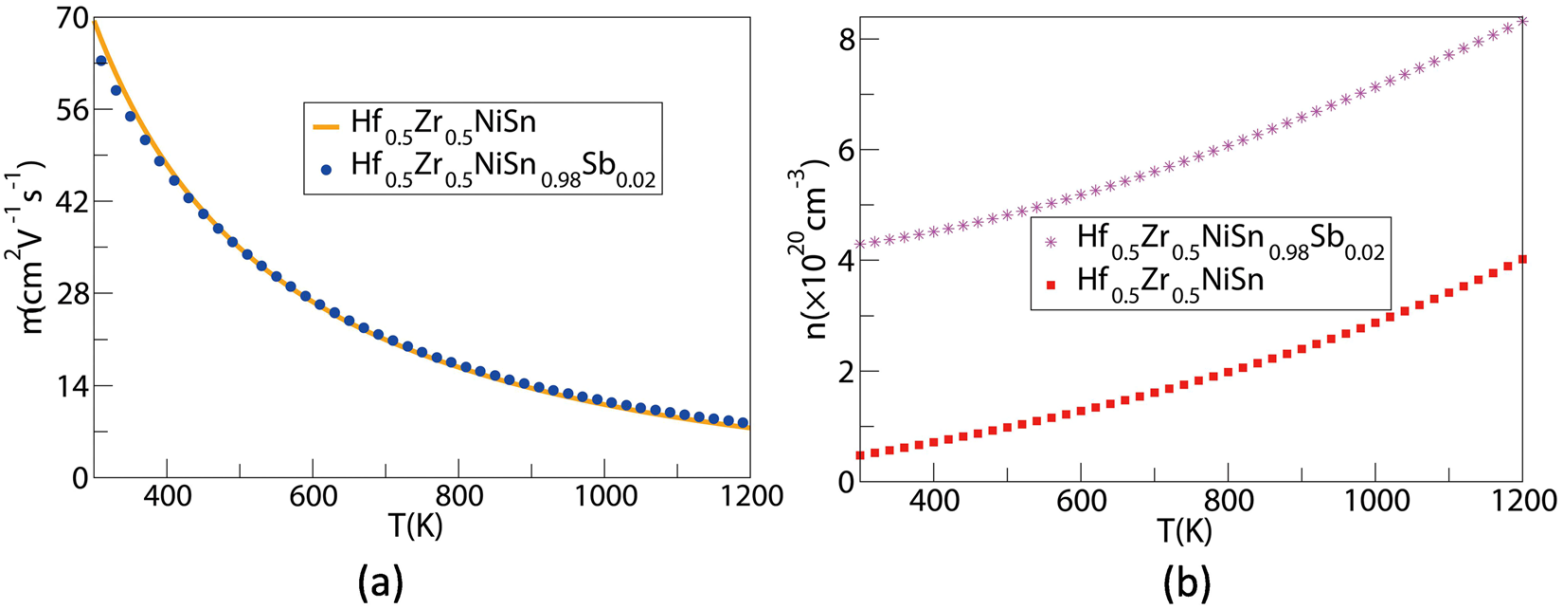
Calculated *μ* (**a**) and *n* (**b**) of Hf_0.5_Zr_0.5_NiSn_1−y_Sb_y_ (y = 0, 0.02) as a function of temperature.

Figure 8(b) manifests that *σ* remarkably enhances, which may originate from the increment of *n* and/or *μ*. To verify this viewpoint, we calculated the temperature dependence of *n* of Hf_0.5_Zr_0.5_NiSn_1−y_Sb_y_ (y = 0, 0.02). *μ* was obtained from the calculated *σ* and *n* by using the expression *μ* = *σ*/*ne*. As seen in Fig. 9, 2% Sb doping really greatly increases *n*, and high *μ* basically remain sametime. Thereby, increasing of *n* may be the main reason for large increase in *σ*. While our calculated *μ* is higher than the experimental value, which may results from two factors. First, our calculated *n* (~4.29 × 10^20^ cm^−3^) is smaller than that of Zhu *et al*.^22^ reported (~5.24 × 10^20^ cm^−3^) at room temperature. High *n* in the experiment may be caused by interstitial Ni atoms. Another potential reason is that the calculated *m* ^*^ (~2.31 *m*_*e*_) is smaller than experimental one (~2.64 *m*_*e*_) in ref.^22^.

To understand the large increase in *n*, the Bader charge analysis of Hf_0.5_Zr_0.5_NiSn and Hf_0.5_Zr_0.5_NiSn_0.98_Sb_0.02_ were calculated by using VASP. The calculated results show that the total number of losing electrons from Sb are 8.87|e| in a unit supercell, and thus *n* is greatly increased. This means that Sb atom loses more electrons than Sn atom, it is one possible reason why Sb doping more effectively increases *n*. Besides, each nearest neighbor Ni atom of Sb averagely obtains more 2.22 e from the Sb atom than that from Sn, manifesting Sb-Ni has a stronger interaction than Sn-Ni. In fact, the stronger hybridization between Sb and Ni atoms will more conduces to increasing the DOS near the CB edge, which is corroborated by Fig. 6(d). This may be another possible reason why Sb doping supplies more electrons. Hence, *σ* remarkably enhances. With increasing *n*, the *E*_F_ shifts deeper in the CB and the differential conductivity turns into more symmetric regarding the Fermi level. In contrast to the dramatical increase of *σ*, *S* reduces. Hf/Sb co-doping reduces *S* from 213 *μ*V K^−1^ (of ZrNiSn) to 153 *μ*V K^−1^ (of Hf_0.5_Zr_0.5_NiSn_0.98_Sb_0.02_) at 800 K, this no explicitly decreasing is most likely due to the *N*_*v*_ increasing which is the key parameters for making a compromise between *n* and *S*.

Calculation results show that Hf_0.5_Zr_0.5_NiSn_0.98_Sb_0.02_ still maintains high *μ* especially above 500 K as revealed in Fig. 9(a) despite the ${m}_{{\rm{b}}}^{\ast }$ increases, which is majorly ascribed to two reasons. For one thing, in general, high *μ* and small ${m}_{{\rm{b}}}^{\ast }$ are found in materials with small Δ*χ* (e.g. IrSb_3_ *μ* = 1320 cm^2^ V^−1^ s^−1^, ${m}_{{\rm{b}}}^{\ast }$ = 0.17 *m*_e_ at 300 K), and low *μ* and large ${m}_{{\rm{b}}}^{\ast }$ are found in ionic materials (e.g. Fe_x_Cr_3−x_Se_4_ *μ* = 0.1 cm^2^ V^−1^ s^−1^, ${m}_{{\rm{b}}}^{\ast }$ = 4 *m*_e_ at 300 K)^43–47^. While, HH compounds, which frequently exhibit both ionic and covalent bonding, bridge these extremes. Thus, Hf_0.5_Zr_0.5_NiSn_0.98_Sb_0.02_ still maintains relatively high *μ* (26.3 cm^2^ V^−1^ s^−1^) with relatively large ${m}_{{\rm{b}}}^{\ast }$ (2.64 *m*_e_) at 300 K. Another, a low Ξ and a low alloy scattering potential with the weak coupling between phonons and electrons are conducive to compensating for the decrease of *μ* due to the large ${m}_{{\rm{b}}}^{\ast }$. This is also a reason for Hf_0.5_Zr_0.5_NiSn_0.98_Sb_0.02_ still maintaining a high *μ*, which has been experimentally confirmed^15–17^. Therefore, the tradeoff between *μ* and ${m}_{{\rm{b}}}^{\ast }$ is achieved, and then maximizing PF through the balance of *σ* and *S* is realized by Eqs (1) and (3). However, the *μ* has a slight reduction below 500 K. This slight reduction can be mainly attributed to the the reduction of *κ*_*l*_ of Hf_0.5_Zr_0.5_NiSn_0.98_Sb_0.02_ with high *n* (~4.29 × 10^20^ cm^−3^ at room temperature) and large ${m}_{{\rm{b}}}^{\ast }$ (~2.31 *m*_e_). As is reported^15,48,49^, acoustic phonon scattering is the dominant scattering mechanism with high *n* and large ${m}_{{\rm{b}}}^{\ast }$ at low temperature. Because when an acoustic phonon wave crosses through the lattice, it induces a local strain in the crystal, resulting in a perturbation of the band and carriers scatterings. The high *μ* retention is due to that the compromise between *μ* and *κ*_*l*_ which is made by minimizing the influence of the acoustic phonon scattering with a small Ξ and a low alloy scattering potential as already mentioned. Thereby, a high TE performance is attained with ~28% enhancement of the highest *ZT* of Hf_0.5_Zr_0.5_NiSn_0.98_Sb_0.02_ compared with Hf_0.5_Zr_0.5_NiSn.

## Conclusions

In summary, the electronic structures and TE transport properties of Hf/Sb co-doping in ZrNiSn have been systematically investigated by using the first-principle calculations and semiclassical Boltzmann theory. We elucidate the possible origins for the improvement of TE properties of ZrNiSn by Hf/Sb co-doping. 50% Hf doping not only increases the *N*_*v*_ with the Γ and M points simultaneously participating in transportation at the bottom of CB but also increases the dispersion of energy bands near the CB edge. These are helpful to increasing *S* and sligtly enhancing *σ* at the same time. Then, 2% Sb co-doping increases total density of states near the *E*_F_ and remains high *μ*, and leads to converging of light and heavy bands. *N*_*v*_ reaches eleven at the conduction band minimum, therefore resulting in a striking improvement in *σ*. Moreover, from the Bader analysis, we also find the reason that why Sb co-doping could provide higher *n*. It results from that Sb loses more electrons and Sb-Ni has a stronger hybridization than Sn-Ni. Our calculation results demonstrate that the *ZT* of Hf_0.5_Zr_0.5_NiSn_0.98_Sb_0.02_ can reach 1.37 with the *n* of 7.56 × 10^18^ cm^−3^ at 1000 K. Thus, Hf/Sb co-doping can be an effective strategy in tuning band structure and enhancing TE properties of ZrNiSn alloy compounds.

### Computational Details

The lattice structures of Hf_x_Zr_1−x_NiSn_1−y_Sb_y_ (x = 0, 0.25, 0.5; y = 0, 0.02) were optimized with the plane-wave cutoff energy of 500 eV and the energy convergence of 10^−6^ eV by the Vienna *ab initio* simulation package (VASP) based on the density functional theory (DFT)^50,51^. The Perdew-Burke-Ernzerh of (PBE) parameterization of generalized- gradient approximation (GGA) was used for the exchange-correlation potential^52^. The full-potential linearized augmented plane wave (FLAPW) method^53^ was applied to calculate the electronic structures of Hf_x_Zr_1−x_NiSn_1−y_Sb_y_ (x = 0, 0.25, 0.5; y = 0, 0.02), which was implemented in WIEN2k^54–56^. Modified Becke-Johnson (mBJ) semi-local exchange potential was employed for improving the accuracy of band gap^57,58^.

Here, the SOC of elements (Hf, Zr, Ni, Sn, and Sb) and the relativistic effect of heavy element Hf were considered. The TE transport properties were evaluated with BoltzTraP code which was based on semiclassical Boltzmann transport theory^59–61^. We used *R*_*mt*_ × *K*_*max*_ = 7 (*K*_*max*_ was the magnitude of the largest *k* vector) as the cutoff parameter. While, *R*_*mt*_, the smallest muffin-tin radius of Ni, Zr, Sn, Hf, and Sb atoms, were set to be 2.45 a.u., 2.42 a.u., 2.47 a.u., 2.46 a.u., and 2.45 a.u., respectively. The rigid band approach (RBA) and the constant scattering time approximation were utilized to evaluate the TE transport properties. When doping, the RBA is supposed to be without changing the band structure of the compound but to shift the Fermi level up or down. The constant scattering time approximation, which is based on a smoothed Fourier interpolation of the bands, is usually implemented for metals and degenerately doped semiconductors. These approximations also have been extensively imposed in the calculation for study many TE materials^62–66^. For Hf_x_Zr_1−x_NiSn alloys (x = 0.25, 0.5), the substitution of Hf atoms at Zr sites was performed in a 12-atoms cell. For Hf_0.5_Zr_0.5_NiSn_0.98_Sb_0.02_ alloy compound, we constructed a 2 × 2 × 3 ZrNiSn supercell, and then replaced Zr atoms with Hf atoms and substituted Sn atom with Sb atom in a 144-atoms cell. To accurately understand the effect of Hf/Sb co-doping on the electronic structures and transport properties of Hf_x_Zr_1−x_NiSn_1−y_Sb_y_ alloy compounds (x = 0, 0.25, 0.5; y = 0, 0.02), we took all kinds of situations as much as possible and the shortest distances among the doping atoms should be as large as possible. The most stable doping site was obtained through optimizing the structures by using the VASP.

## Electronic supplementary material

Supplementary materials
