# Supplementary material for: Hf/Sb co-doping induced a high thermoelectric performance of ZrNiSn: First-principles calculation
Source: Sci Rep. 2017 Nov 6;7:14590. doi: 10.1038/s41598-017-15205-y (PMC5674061; doi:10.1038/s41598-017-15205-y)
Supplement: Supplementary file 1 — Supplementary materials [file 41598_2017_15205_MOESM1_ESM.pdf]

Supplementary Materials for

Hf/Sb co-doping induced a high thermoelectric performance of ZrNiSn: First-principles calculation

Ju Zhang, Xiwen Zhang, Yuanxu Wang*

Institute for Computational Materials Science,

School of Physics and Electronics, Henan University,

Kaifeng 475004, People's Republic of China

(Dated: September 15, 2017)

* E-mail: wangyx@henu.edu.cn

The half-Heusler ZrNiSn and HfNiSn have the cubic MgAgAs-type structure and space group of $F\bar{4}3m$. The unit cell is crystallized as four interpenetrating face-centered cubic (FCC) sublattices. Optimized lattice constants of ZrNiSn and HfNiSn are $\sim$ 6.141 Å and 6.113 Å, respectively, which agree well with the experimental values ($\sim$ 6.110 Å and 6.066 Å).[1]

We calculated the band structures of ZrNiSn and HfNiSn using the first-principles method with the modified Becke-Johnson semi-local exchange potential as implement in the WIEN2k. The calculated band structures of half-Heusler ZrNiSn and HfNiSn are shown in Fig. 1. The band gaps are 0.51 eV for ZrNiSn and 0.32 eV for HfNiSn, which are in good agreement with previous theoretical calculations values.[2] The calculated band gaps are larger than those experimental values ($\sim$ 0.18 eV and 0.22 eV, respectively).[3] This is mainly caused by the formation of impurity band in the gap of synthesized compounds, due to the appearance of Ni interstitial atoms with its d orbitals when Ni atoms excess (>25%).[4] The band degeneracy of ZrNiSn is six at the Γ point of the top of valence band (VB). While, different from ZrNiSn, the bands of HfNiSn at the top of split due to spin-orbital coupling effect. However, Zou *et al.* [2] reported that the bands at the top of VB are degenerated, because they did not consider spin-orbital couping effect. Thus, we include spin-orbital coupling effect in our calculations for these compounds.

[1] Öğüt, S. & Rabe, K., Band gap and stability in the ternary intermetallic compounds NiSnM (M=Ti,Zr,Hf): A first-principles study. *Phys. Rev. B* **51**, 10443-10453 (1995).

[2] Zou, D., Xie, S., Liu, Y., Lin, J., & Li, J., Electronic structure and thermoelectric properties of half-Heusler $Zr_{0.5}Hf_{0.5}NiSn$ by first-principles calculations. *J. Appl. Phys.* **113**, 193705 (2013).

[3] Aliev, F. *et al.*, Gap at the Fermi level in the intermetallic vacancy system RBiSn (R = Ti, Zr, Hf). *Z. Phys. B: Condens. Matter* **75**, 167-171 (1989).

[4] Romaka, V. *et al.*, Peculiarities of structural disorder in Zr- and Hf-containing Heusler and half-Heusler stannides. *Intermetallics* **35**, 45-52 (2013).

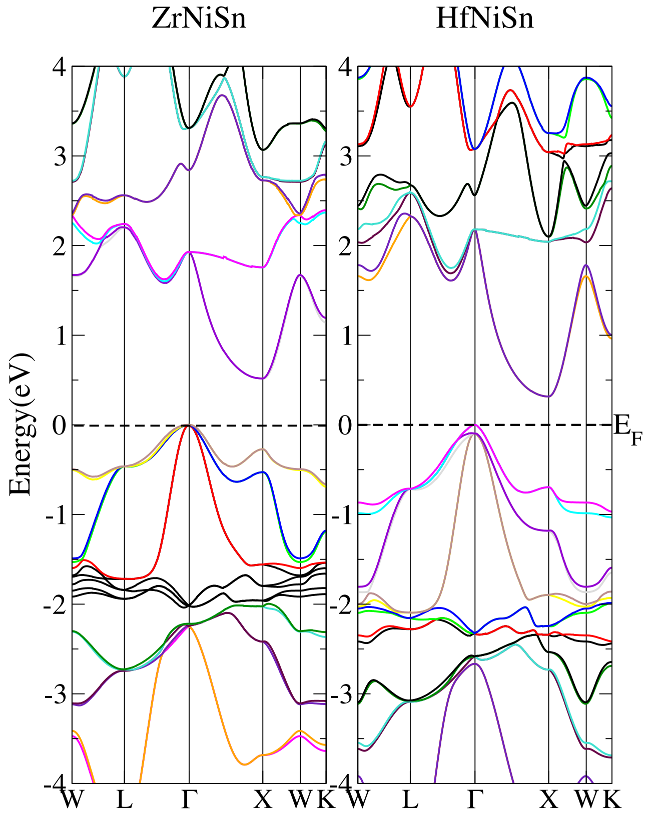

FIG. 1: The band structures with spin-orbital coupling of half-Heusler ZrNiSn and HfNiSn with the space group of $F\bar{4}3m$. The Fermi level is set to be zero.
